# Polypharmacy as an ordered indicator of therapeutic complexity in a national cardiovascular prevention programme

**DOI:** 10.3389/fphar.2026.1806974

**Published:** 2026-04-21

**Authors:** Jacek Gonos, Marek Tradecki, Grzegorz Kubielas, Ercole Vellone, Olga Fedorowicz, Michał Czapla, Bartosz Uchmanowicz

**Affiliations:** 1 Basic Health Care Unit, Foundation of Wroclaw Medical University, Wrocław, Poland; 2 Department of Environmental Health, Occupational Medicine and Epidemiology, Faculty of Health Sciences, Wroclaw Medical University, Wroclaw, Poland; 3 Medical Boards of the Social Insurance Institution, Wroclaw, Poland; 4 Department of Nursing, Faculty of Nursing and Midwifery, Wroclaw Medical University, Wroclaw, Poland; 5 Department of Health Care Services, Polish National Health Fund, Central Office in Warsaw, Warsaw, Poland; 6 Department of Biomedicine and Prevention, University of Rome Tor Vergata, Rome, Italy; 7 Department of Clinical Pharmacology, Faculty of Pharmacy, Wroclaw Medical University, Wroclaw, Poland; 8 Division of Scientific Research and Innovation in Emergency Medical Service, Department of Emergency Medical Service, Faculty of Nursing and Midwifery, Wroclaw Medical University, Wroclaw, Poland; 9 Group of Research in Care (GRUPAC), Faculty of Health Science, University of La Rioja, Logroño, Spain; 10 Nursing Care and Education Research Group (GRIECE), Faculty of Nursing and Podology, University of Valencia, Valencia, Spain

**Keywords:** cardiovascular diseases, middle aged, multimorbidity, polypharmacy, primary health care

## Abstract

**Background:**

The prevalence of polypharmacy has been rising worldwide, most studies focuses on older adults. However, little is known about patterns and determinants of polypharmacy in middle-aged individuals engaged in structured national prevention programmes. This exploratory study examines how clinical characteristics, comorbidity structure, and system-level actions relate to increasing medication burden in participants of the Polish Prophylaxis 40 PLUS prevention programme, which focuses on the prevention and early detection of lifestyle-related (civilization) diseases, particularly cardiovascular conditions.

**Methods:**

A cohort of 151 adults aged ≥40 years was stratified into three ordinal polypharmacy categories (5, 6, ≥7 drugs). Patients demographics, lifestyle behaviours, blood-pressure measures, ICD-10 comorbidity blocks, and indicators of medical actions were analysed. Ordinal proportional-odds regression (forward and backward stepwise selection) was performed with multimorbidity included as an adjustment covariate. Bootstrap resampling (B = 200) assessed parameter stability.

**Results:**

Circulatory system diseases (ICD-10 I) were consistently associated with higher odds of belonging to a higher polypharmacy category (OR 2.3–2.6). Normalisation of previously irregular heart rhythm strongly predicted higher medication burden (OR 6.0–8.4). Sensory system diseases (ICD-10 H) were also positively associated with polypharmacy, whereas nervous-system diseases (ICD-10 G) showed an inverse relationship. Higher baseline diastolic blood pressure was negatively associated with medication count (OR 0.93–0.95 per mmHg). Educational attainment demonstrated a weaker, exploratory positive association. Bootstrap analysis confirmed the robustness of the main predictors (ICD-10 I, ICD-10 G/H, heart-rhythm change, baseline DBP).

**Conclusion:**

In middle-aged adults within a Polish national prevention programme, polypharmacy reflects treatment intensity and system-level complexity rather than multimorbidity alone. Cardiovascular disease, rhythm-control interventions, and multi-specialty care were associated with higher medication burden. These findings highlight the importance of integrating structured medication review, targeted deprescribing, and equitable preventive pharmacotherapy into cardiovascular prevention pathways.

## 1 Introduction

Cardiovascular diseases (CVD) remain the leading cause of premature mortality and disability worldwide, despite substantial progress in prevention and treatment over recent decades (Roth et al., 2020; Banach et al., 2025). Contemporary prevention frameworks emphasise structured lifetime risk assessment, early identification of modifiable risk factors, and comprehensive intervention on blood pressure, lipids, smoking, body weight, and lifestyle behaviours (Visseren et al., 2021; Lloyd-Jones et al., 2022; Howard et al., 2024). The 2021 European Society of Cardiology (ESC) prevention guidelines and the American Heart Association’s Life’s Essential 8 (LE8) construct highlight the need to move beyond single risk factors towards integrated cardiovascular health, combining traditional metrics such as blood pressure and cholesterol with health behaviours, diet, and sleep (Visseren et al., 2021; Lloyd-Jones et al., 2022; Howard et al., 2024). In parallel, advances in pharmacotherapy—particularly statins, combination lipid-lowering regimens, and intensive blood pressure control—have transformed the prevention landscape (Banach et al., 2025; Badimon et al., 2023). Multinational registry data, such as EUROASPIRE V, show that secondary prevention remains suboptimal in many patients, but also illustrate the central role of pharmacological treatment in achieving guideline targets (Kotseva et al., 2019; Marques-Vidal et al., 2025).

National preventive programmes are one of many ways to operationalise these recommendations at scale. In Poland, Profilaktyka 40 PLUS (Prophylaxis 40 PLUS) is a nationwide programme offering adults aged 40 years and older free access to standardised health assessment laboratory tests, and risk-factor screening (Ministerstwo Zdrowia). The programme aims to improve early detection of cardiometabolic risk, facilitate access to primary and specialist care, and support implementation of ESC-aligned preventive strategies in routine practice (Visseren et al., 2021; Ministerstwo Zdrowia). Participants undergo structured evaluation in primary care and receive feedback on cardiovascular risk. They are offered tailored counselling and, where indicated, pharmacological therapy. The present study focuses on a subset of middle-aged adults recruited within this Prophylaxis 40 PLUS framework.

Within such a preventive context, polypharmacy most commonly defined as the concurrent use of five or more medications has become a key but ambivalent phenomenon (Masnoon et al., 2017). On the one hand, guideline-directed preventive therapy often requires combinations of antihypertensive, lipid-lowering, antiplatelet or anticoagulant, and glucose-lowering agents, particularly in individuals with multiple conditions (Banach et al., 2025; Visseren et al., 2021; Marques-Vidal et al., 2025). On the other hand, polypharmacy is associated with increased risk of adverse drug reactions, drug–drug and drug–disease interactions, falls, hospitalisations, functional decline, and mortality, especially among older and multimorbid patients (Guillot et al., 2020; Maher et al., 2014; Davies et al., 2020; Khezrian et al., 2020). Reviews consistently show that higher medication counts are linked to poor outcomes and healthcare utilisation, but also stress that the underlying mechanisms are complex and context-dependent (Maher et al., 2014; Davies et al., 2020; Khezrian et al., 2020). In response, the concept of appropriate versus inappropriate polypharmacy has been proposed, underlining that not all polypharmacy is harmful if it reflects evidence-based, person-centred care (Pazan and Wehling, 2021).

Epidemiological data indicate that polypharmacy prevalence has increased significantly in recent decades. Systematic reviews and meta-analyses report that between one-quarter and more than half of older adults worldwide receive five or more drugs, with considerable variation by setting and definition (Delara et al., 2022; Nicholson et al., 2024; Januário et al., 2023). National and regional studies similarly document rising trends in polypharmacy and hyper-polypharmacy (e.g., ≥10 drugs) over time (Cho et al., 2022; Kardas et al., 2021; Dovjak, 2022; Nishtala and Salahudeen, 2015). In Poland, analysis of a real-world database including nearly 38 million citizens showed a high and age-dependent burden of polypharmacy, with particularly high exposure in older adults and in those with chronic conditions (Kardas et al., 2021). Comparable longitudinal trends have been described in New Zealand and other high-income countries, with increasing use of preventive and symptomatic medications over 9–10-year periods (Dovjak, 2022). These patterns underline that polypharmacy has become the norm rather than the exception in many health systems.

Polypharmacy is tightly interwoven with multimorbidity, typically defined as the presence of two or more chronic conditions. Multimorbidity affects a growing proportion of adults and is especially prevalent in middle-aged and older populations (Skou et al., 2022). It is associated with poorer quality of life, functional decline, and increased healthcare utilisation, and strongly predicts the need for multiple medications (Skou et al., 2022). Hospital-based studies show that the majority of older inpatients live with both multimorbidity and polypharmacy, and that this combination is associated with higher risk of adverse outcomes and complex discharge planning (Zhao et al., 2023). Community-based work has linked polypharmacy and multimorbidity to greater risk of medication-related problems, including potentially inappropriate prescribing and drug interactions (Ye et al., 2022). Together, this evidence suggests that polypharmacy should be interpreted as a system-level marker of multimorbidity and care intensity rather than a simple count of pills (Masnoon et al., 2017; Guillot et al., 2020; Skou et al., 2022).

Managing polypharmacy safely and effectively is therefore a major clinical and organisational challenge. Scoping reviews have catalogued numerous tools and strategies—from explicit criteria lists to clinical decision-support systems and multidisciplinary review models—aimed at improving prescribing in older adults with multiple conditions (Kurczewska-Michalak et al., 2021). Nurses and other frontline professionals play a crucial role in medication reconciliation, adherence support, and patient education in this context (Kim and Parish, 2017). Epidemiological studies show that higher medication counts are linked to adverse outcomes even in relatively robust older populations, reinforcing the need for structured review processes (Gnjidic et al., 2012). Narrative reviews and consensus documents emphasise that polypharmacy should not be reduced to a numerical threshold but considered within a broader framework of appropriateness, goals of care, and patient preferences (Pazan and Wehling, 2021; Kim and Parish, 2017; Pazan and Wehling, 2017). Interventional evidence is emerging: Cochrane and other systematic reviews suggest that medication reviews, pharmacist-physician collaboration, and structured deprescribing can reduce potentially inappropriate prescribing, although effects on harder outcomes such as mortality or hospitalisation are more modest and heterogeneous (Ye et al., 2022; Rankin et al., 2018; Jungo et al., 2023). Recent cluster-randomised trials in primary care, such as the OPTICA study, have further tested structured medication-optimisation strategies in multimorbid patients, with mixed but encouraging results regarding prescribing quality and safety (Rankin et al., 2018). Multidisciplinary clinic models dedicated to polypharmacy management also show promise in improving specific outcomes and aligning treatment with patient priorities (Roncal-Belzunce et al., 2024; Roncal‐Belzunce et al., 2025).

Despite this body of work, important gaps remain. First, most polypharmacy research focuses on older adults (commonly ≥65 years) in geriatric, hospital or long-term-care settings, while much less is known about middle-aged adults who are at the beginning of potentially decades-long exposure to multiple drugs (Delara et al., 2022; Nicholson et al., 2024; Januário et al., 2023; Cho et al., 2022; Skou et al., 2022; Wang et al., 2024). Second, relatively few studies have examined polypharmacy within organised cardiovascular prevention programmes such as Prophylaxis 40 PLUS, where preventive therapy is actively promoted and facilitated at a population level. Third, the interaction between polypharmacy, multimorbidity, and downstream outcomes in these settings is likely influenced by digital health tools, risk communication, and behaviour-change interventions, which are increasingly incorporated into prevention strategies (Visseren et al., 2021; Lloyd-Jones et al., 2022; Marques-Vidal et al., 2025; Widmer et al., 2015; Varma et al., 2021). Finally, adverse lifestyle exposures such as smoking remain important contextual drivers of both cardiovascular risk and pharmacotherapy intensity (Roth et al., 2020; Jha and Peto, 2014).

In this exploratory sub-study based on a Prophylaxis 40 PLUS-derived cohort of middle-aged adults with multimorbidity, we therefore focus specifically on polypharmacy as an outcome. We use an ordinal classification of concurrent drugs (≤5, 6, ≥7 medications) and apply a proportional-odds regression framework to explore how demographic factors, lifestyle behaviours, blood-pressure measures, ICD-10-based comorbidity blocks, and indicators of medical action (e.g., heart-rhythm normalisation) align with increasing medication burden, adjusting for overall multimorbidity. By treating polypharmacy as an ordered, system-level indicator rather than a simple dichotomy, we aim to: (1) characterise the structure of pharmacotherapy complexity in middle-aged adults participating in a national cardiovascular prevention programme, and (2) identify patient- and system-related factors that might guide more targeted medication review and deprescribing strategies in future iterations of such programmes.

## 2 Methodology

### 2.1 Study design

The study adopted a retrospective observational design and was based on data from participants of the national preventive programme “Prophylaxis 40 PLUS”, delivered through primary healthcare clinics in the Lower Silesian region of Poland. The analysed data covered the period from January 2021 to December 2024. The study was conducted and reported in accordance with the STROBE (Strengthening the Reporting of Observational Studies in Epidemiology) recommendations.

### 2.2 Study population

**FIGURE 1:**
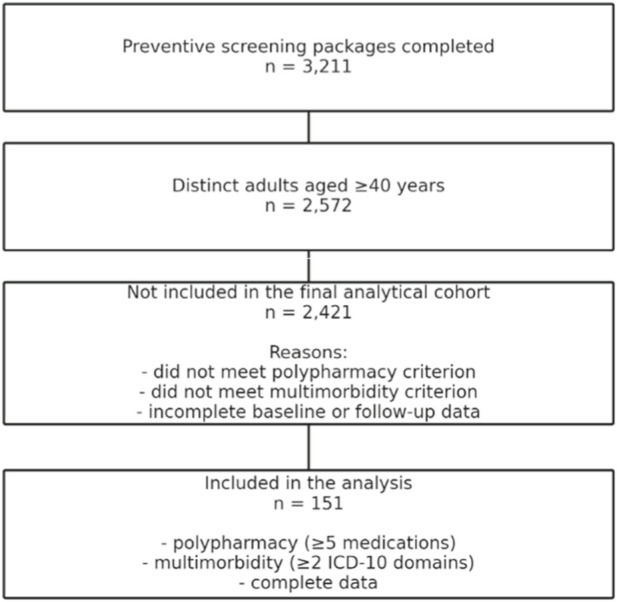
Participant flow diagram of the study sample selection.

Study sample originated from participants enrolled in the national preventive programme “Prophylaxis 40 PLUS”, implemented in primary healthcare settings in the Lower Silesian region of Poland. Over the study period, 3,211 preventive screening packages were completed by 2,572 distinct adults aged 40 years and older, as the programme allowed repeated participation after a 12 months interval (Figure 1).

For the purposes of the present analysis, a subgroup of 151 participants was selected. The final analytical cohort comprised 151 participants who fulfilled all eligibility criteria and had complete baseline and follow-up data within the programme database. Inclusion was limited to individuals meeting criteria for both polypharmacy, defined as the regular use of five or more prescribed medications, and multimorbidity, defined as the presence of at least two chronic conditions classified according to the International Classification of Diseases, 10th Revision. Middle age was defined as 40–59 years; however, due to the structure of the available data, the final analytical cohort included participants aged 40–53 years. Specifically, participants older than 53 years did not simultaneously fulfil both the polypharmacy and multimorbidity inclusion criteria within the available dataset, which explains the discrepancy between the programme’s nominal age range (40–59 years) and the observed range of the final analytical sample (40–53 years). This limitation is acknowledged in the Study Limitations section.

Eligibility further required complete clinical and laboratory data from both baseline and follow up assessments, enabling calculation of the SCORE2 cardiovascular risk index. Participants with incomplete records were excluded from the analysis. All individuals participated voluntarily and were managed according to standard national procedures governing the preventive programme.

### 2.3 Assessment of pharmacotherapy and medication-related variables

Pharmacotherapy was assessed using electronic medical records obtained within the Prophylaxis 40 PLUS programme at baseline and follow up visits. For each participant, all regularly prescribed systemic medications documented at the time of assessment were recorded. Medications intended solely for use needed basis, short term antibiotic therapies, and over the counter supplements were excluded. Each active pharmacological agent was counted as a single medication, irrespective of dose or formulation.

For the purposes of this study, polypharmacy was defined as the concurrent use of ≥5 prescribed medications. To reflect increasing levels of pharmacotherapy complexity, medication burden was operationalized as an ordinal variable. Based on the observed distribution of medication counts in the study population and commonly applied thresholds in the literature, participants were classified into three ordered categories: 5 medications, 6 medications, and ≥7 medications. This categorization was designed to distinguish between lower and higher degrees of polypharmacy and to enable the analysis of graded associations with clinical and healthcare system related factors.

Medications were further categorized according to their primary therapeutic indication, with particular emphasis on cardiovascular pharmacotherapy, including antihypertensive agents, lipid lowering medications, antiplatelet and anticoagulant therapies, antiarrhythmic drugs, and glucose lowering agents. The total number of concurrently prescribed medications served as a summary indicator of medication burden.

To account for underlying disease complexity, multimorbidity was assessed independently using diagnostic data coded according to the International Classification of Diseases, 10th Revision. The presence of diagnoses across ICD 10 chapters was recorded, and a multimorbidity count variable was derived to represent the number of coexisting disease domains. This variable was included as an adjustment covariate in all multivariable analyses to separate medication burden related to therapeutic decision making from that attributable to disease burden alone.

In addition to disease related factors, several medication related clinical actions were identified as indicators of healthcare system level management. These included initiation of new pharmacological treatment, modification of existing dosages, management of recurrent conditions, referral to specialist care, and changes in heart rhythm status between visits. These variables were analysed as markers of therapeutic decisions that could influence medication burden independently of baseline morbidity.

Polypharmacy was treated as a structured outcome reflecting both patient level clinical needs and healthcare system responses rather than as a direct proxy for inappropriate prescribing. Proportional odds ordinal logistic regression models were applied to examine associations between progressively higher levels of medication use and selected cardiovascular diagnoses, blood pressure parameters, rhythm control outcomes, and non cardiovascular disease categories, with adjustment for multimorbidity.

### 2.4 Statistical methods

All analyses were performed in R 4.5.0 using the packages readr, dplyr, magrittr, openxlsx, ggplot2, MASS, and, when available, brant. Python 3.13.3 (package: matplotlib) was used for visualization. The workflow was fully scripted and reproducible, covering all stages from data import to model diagnostics and bootstrap validation.

The analytical goal was to explore patterns of co-occurrence between patient characteristics, comorbidity structure, and increasing levels of polypharmacy, rather than to infer unidirectional causal relationships. This “reverse modelling” perspective treats polypharmacy as a composite endpoint reflecting both disease burden and the dynamics of care, allowing the model to capture how clinical and system-level factors align along the drug-count gradient.

#### 2.4.1 Patient-level descriptive analyses

Baseline characteristics were summarized across three ordinal polypharmacy categories (≤5, 6, ≥7 drugs). Continuous variables are presented as median (1st–3rd quartile) and compared using the Jonckheere–Terpstra test for ordered trends; Hodges–Lehmann estimates (95% CI) were used to quantify median shifts between the lowest and highest categories. Categorical variables are shown as counts and percentages, with the Cochran–Armitage trend test applied for ordinal data and Pearson’s χ^2^ or Fisher’s exact test for nominal data. This step provided a structured overview of population characteristics along the polypharmacy gradient to inform subsequent model specification.

#### 2.4.2 Multivariate modeling

**FIGURE 2:**
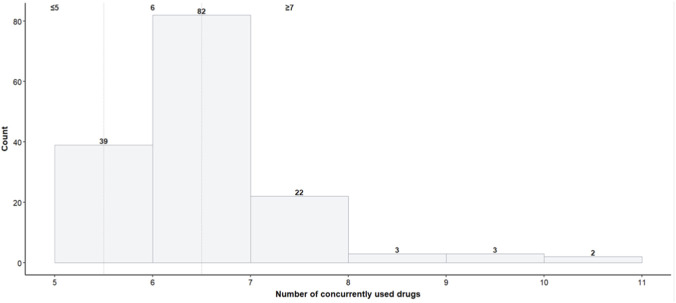
Distribution of the modelled variable—the count of concurrently administered drugs.

We first generated a simple indicator of multimorbidity from ICD-10 chapter flags. Each ICD code was translated into a binary presence variable, and a count variable (multicomorbidity_count) was created to summarize overall comorbidity. Only after this aggregation were rare ICD categories (1-2 occurrences) excluded from modelling to avoid instability and separation issues typical for small samples. The dependent variable was the total number of concurrently used drugs, categorized into three ordered levels: ≤5 (“5”), 6 (“6”), and ≥7 (“7plus”). This ordinal formulation was guided by the data distribution (Figure 2) and by the pragmatic clinical view that higher medication counts represent qualitatively distinct states of therapeutic complexity.

To examine whether system-level decisions added explanatory value beyond individual characteristics, two datasets were defined:

- Model A, containing patient-level variables (demographics, anthropometrics, baseline and change markers, and the retained ICD indicators);
- Model B, extending Model A with system-level descriptors of medical actions (e.g., initiation of new therapy, referrals, diagnostic expansion, dietary recommendations).

Continuous variables were median-centered to improve numerical stability, low-cardinality variables were coerced to factors, and collinear predictors were pruned via correlation-based thinning (r > 0.9).

Given the modest sample size, we applied an explicitly exploratory approach based on proportional odds logistic regression. Two complementary selection strategies were used:

- Forward selection (entry threshold p < 0.05), emphasizing parsimonious, data-driven inclusion;
- Backward selection (removal threshold p ≥ 0.10), emphasizing retention of variables with at least modest evidence once others are accounted for.

In both schemes, the multimorbidity count could be forced into the model to ensure clinical interpretability. Every modelling step—including variable entry, removal, and the associated p-values—was logged automatically to maintain analytical transparency. Each final model was evaluated with standard goodness-of-fit metrics (log-likelihood, likelihood-ratio test, AIC, BIC, McFadden’s pseudo-R^2^). The proportional odds assumption was assessed for all ordinal logistic regression models using the Brant-type omnibus and component-wise χ^2^ tests. In the forward selection models (A and A with forced multimorbidity adjustment), omnibus p-values of 0.42 and 0.57 indicated no departure from proportionality. In the backward selection framework, the omnibus test reached formal significance (p < 0.001), driven almost exclusively by the variable ‘change in FG’ (χ^2^ = 16.22, p < 0.001, see Supplementary Material), which showed a pronounced monotonic trend across increasing polypharmacy categories. All other predictors remained well within proportionality limits. Given the small sample size (N = 151) and the fact that the proportionality breach was limited to a single term with coherent directionality, no partial proportional odds model was introduced. Instead, results were interpreted with due caution, emphasizing robustness over overfitting corrections.

To assess the stability of model parameters under resampling, all final models (forward and backward, with and without forced terms) were subjected to nonparametric bootstrap (B = 200). For each coefficient—including intercept cutpoints separating outcome categories—bootstrap means, standard deviations, and percentile intervals (2.5%–97.5%) were computed. This procedure quantifies the robustness of the identified associations without implying model selection certainty. Supplementary Material features all analytical steps executed within one consistent pipeline from raw data processing and feature engineering (Supplementary Table S1) to model fitting (Supplementary Tables S2-S3), diagnostics of model assumptions (Brant tests, Supplementary Table S4), overall goodness-of-fit metrics (Supplementary Table S5), and bootstrap estimation (Supplementary Table S6). This information serves as additional documentation behind multivariate inference found in the main body of this manuscript.

## 3 Results

### 3.1 Population sample characteristics

The study cohort included 151 patients, stratified by the number of concurrently prescribed drugs into three ordered categories: ≤5 drugs (n = 39), 6 drugs (n = 82), and ≥7 drugs (n = 30). Table 1 presents the distribution of continuous variables and Table 2 summarizes categorical characteristics across these strata.

**TABLE 1:** Univariate characteristics of the population sample in context of polypharmacy - quantitative variables.

Variable	Number of concurrent drugs			HL 5 drugs vs. ≥ 7 drugs	JT p
	5 (N = 39)	6 (N = 82)	≥7 (N = 30)		
Age [years]	47.0 (44.0–49.5)	48.0 (44.2–51.0)	49.0 (46.0–51.0)	−1.00 (−3.00 to 1.00)	0.224
BMI [kg/m^2^]	28.0 (24.2–30.6)	28.1 (25.0–31.8)	26.0 (22.9–28.5)	1.20 (−1.42–3.43)	0.462
Body mass [kg]	71.0 (65.0–91.0)	79.5 (71.0–93.8)	70.0 (62.8–80.5)	4.00 (−3.00–13.00)	0.363
Height [cm]	168.0 (160.0–176.0)	166.5 (162.0–174.8)	164.0 (160.5–169.8)	3.00 (−2.00–8.00)	0.230
Waist circumference [cm]	91.0 (82.5–104.0)	95.0 (88.0–106.0)	91.0 (81.0–101.0)	2.00 (−6.00–9.00)	0.823
Baseline SBP [mmHg]	135.0 (122.0–146.5)	137.0 (128.0–147.8)	133.5 (121.2–145.0)	2.00 (−8.00–13.00)	0.735
Baseline DBP [mmHg]	90.0 (80.0–95.0)	87.0 (80.0–92.8)	83.0 (73.5–90.8)	6.00 (−1.00–12.00)	0.095
SBP at follow-up [mmHg]	131.0 (121.0–138.5)	131.5 (126.0–138.0)	131.5 (124.5–135.0)	0.00 (−6.00 to 5.00)	0.823
DBP at follow-up [mmHg]	82.0 (78.5–86.5)	82.0 (77.0–85.0)	80.0 (76.2–84.8)	2.30 (−1.00–6.00)	0.132
Change in SBP [mmHg]	−3.0 (−13.0–5.5)	−6.0 (−13.0–3.0)	−5.0 (−10.0–5.8)	−1.00 (−8.00 to 5.00)	0.736
Change in DBP [mmHg]	−5.0 (−12.5–0.0)	−5.0 (−9.0–1.0)	−3.0 (−10.2–4.8)	−3.00 (−8.00 to 2.00)	0.172
Baseline non-HDLc [mg/dL]	154.0 (136.5–176.0)	150.0 (124.2–169.2)	148.5 (109.5–177.8)	5.00 (−16.00–26.00)	0.611
Non-HDLc at follow-up [mg/dL]	137.0 (119.5–155.5)	142.0 (123.8–160.5)	127.0 (111.2–158.8)	7.00 (−11.00–24.00)	0.611
Change in non-HDLc [mg/dL]	−11.0 (−23.5–6.0)	−8.0 (−17.0–3.8)	−14.5 (−27.5–2.0)	3.00 (−7.00–12.00)	0.710
Baseline TG [mg/dL]	108.0 (71.0–171.0)	140.0 (96.0–193.5)	114.5 (101.5–161.5)	−14.00 (−45.00 to 18.00)	0.392
TG at follow-up [mg/dL]	110.0 (72.0–152.5)	137.5 (97.0–181.5)	119.5 (85.8–155.0)	−3.54 (−31.00 to 20.00)	0.590
Change in TG [mg/dL]	−12.0 (−36.5–15.5)	−8.0 (−31.2–24.0)	−17.0 (−36.5–8.8)	8.00 (−14.00–28.00)	0.510
Baseline FG [mg/dL]	95.0 (91.0–112.0)	96.0 (87.2–104.8)	95.5 (87.0–106.0)	3.00 (−5.00–9.00)	0.391
FG at follow-up [mg/dL]	95.0 (88.0–99.0)	93.5 (86.2–99.0)	92.5 (87.2–99.0)	0.00 (−4.00–5.00)	0.964
Change in FG [mg/dL]	−5.0 (−13.5–3.0)	−5.0 (−10.8–3.0)	−4.0 (−9.0–3.5)	−2.00 (−8.00 to 3.00)	0.386
Baseline SCORE2 [%]	3.0 (2.0–5.0)	3.0 (2.0–6.0)	4.0 (2.0–6.0)	∼0.00 (−2.00 to 1.00)	0.586
SCORE2 at follow-up [%]	3.0 (2.0–5.0)	3.0 (2.0–5.0)	3.0 (2.0–4.0)	∼0.00 (−1.00 to 1.00)	0.814
Change in SCORE2 [pp]	0.0 (−1.5–0.0)	0.0 (−1.0–0.0)	−1.0 (−2.0–0.0)	∼0.00 (∼0.00–1.00)	0.284
Multicomorbidity [n concurrent ICD-10 categories]	4.0 (4.0–4.5)	4.0 (4.0–4.0)	4.0 (4.0–4.0)	-	0.983

Values are given as median value and 1st–3rd quartile; the HL (Hodges-Lehmann estimate) is given with its 95% CI. JT, Jonckheere-Terpstra test.

**TABLE 2:** Univariate characteristics of the population sample in context of polypharmacy—qualitative variables.

Variable domain	Variable	Category	Overall (N = 151)	Number of concurrent drugs			p
				5 (N = 39)	6 (N = 82)	≥7 (N = 30)	
Demography and socioeconomic status	Year of enrolment	2021	3 (2.0)	3 (7.7)	0 (0.0)	0 (0.0)	**0.031**
		2022	72 (47.7)	17 (43.6)	41 (50.0)	14 (46.7)	
		2023	64 (42.4)	18 (46.2)	31 (37.8)	15 (50.0)	
		2024	12 (7.9)	1 (2.6)	10 (12.2)	1 (3.3)	
	Sex: male	Yes	51 (33.8)	14 (35.9)	31 (37.8)	6 (20.0)	0.200
	Domicile: rural (village)	Yes	69 (45.7)	21 (53.8)	38 (46.3)	10 (33.3)	0.234
	Education	Primary	3 (2.0)	0 (0.0)	2 (2.4)	1 (3.3)	0.344
		Secondary/trade	109 (72.2)	33 (84.6)	56 (68.3)	20 (66.7)	
		Higher	39 (25.8)	6 (15.4)	24 (29.3)	9 (30.0)	
	Education (aggregated)	Primary/secondary/trade	112 (74.2)	33 (84.6)	58 (70.7)	21 (70.0)	0.141
		Higher	39 (25.8)	6 (15.4)	24 (29.3)	9 (30.0)	
	Civil status: not married	Yes	23 (15.2)	7 (17.9)	8 (9.8)	8 (26.7)	0.076
Health-related habits	Smoking habit at baseline	Yes	55 (36.4)	18 (46.2)	24 (29.3)	13 (43.3)	0.134
	Gave up smoking at follow-up	Yes	11 (7.3)	3 (7.7)	6 (7.3)	2 (6.7)	0.987
	Alcohol consumption frequency at baseline	None/less than several times a year	33 (21.9)	5 (12.8)	22 (26.8)	6 (20.0)	0.399
		Several times a year	69 (45.7)	19 (48.7)	34 (41.5)	16 (53.3)	
		Several times a month	41 (27.2)	13 (33.3)	20 (24.4)	8 (26.7)	
		Several times a week	8 (5.3)	2 (5.1)	6 (7.3)	0 (0.0)	
	Alcohol consumption frequency at follow-up	None/less than several times a year	40 (26.5)	6 (15.4)	28 (34.1)	6 (20.0)	0.227
		Several times a year	88 (58.3)	25 (64.1)	43 (52.4)	20 (66.7)	
		Several times a month	20 (13.2)	6 (15.4)	10 (12.2)	4 (13.3)	
		Several times a week	3 (2.0)	2 (5.1)	1 (1.2)	0 (0.0)	
	Reduced alcohol consumption frequency at follow-up	Yes	37 (24.5)	9 (23.1)	24 (29.3)	4 (13.3)	0.215
	Coffee-drinking habit at baseline	Yes	144 (95.4)	38 (97.4)	79 (96.3)	27 (90.0)	0.165
	Coffee-drinking habit at follow-up	Yes	144 (95.4)	38 (97.4)	79 (96.3)	27 (90.0)	
	Changed coffee-drinking habit at follow-up	no	151 (100.0)	39 (100.0)	82 (100.0)	30 (100.0)	-
	Drinks coffee without milk at baseline	Yes	62 (42.8)	15 (39.5)	33 (41.2)	14 (51.9)	0.350
	Drinks coffee without milk at follow-up	Yes	62 (42.8)	15 (39.5)	33 (41.2)	14 (51.9)	
Heart rhythm, and blood pressure status — new and old recommendations	Regular HR at baseline	Yes	20 (13.2)	5 (12.8)	6 (7.3)	9 (30.0)	**0.007**
	Irregular HR at follow-up	Yes	6 (4.0)	3 (7.7)	2 (2.4)	1 (3.3)	0.377
	Irregular-to-regular HR change at follow-up	Yes	14 (9.3)	2 (5.1)	4 (4.9)	8 (26.7)	**0.001**
	Regular-to-irregular HR change at follow-up	no	151 (100.0)	39 (100.0)	82 (100.0)	30 (100.0)	**<0.001**
	Baseline SBP category (new recommendations)	Normotension	23 (15.2)	8 (20.5)	9 (11.0)	6 (20.0)	0.598
		High-normal	62 (41.1)	16 (41.0)	34 (41.5)	12 (40.0)	
		Hypertension	66 (43.7)	15 (38.5)	39 (47.6)	12 (40.0)	
	Baseline SBP category (old recommendations)	Normotension	53 (35.1)	14 (35.9)	25 (30.5)	14 (46.7)	0.474
		High-normal	32 (21.2)	10 (25.6)	18 (22.0)	4 (13.3)	
		Hypertension	66 (43.7)	15 (38.5)	39 (47.6)	12 (40.0)	
	Baseline DBP category (new recommendations)	Normotension	11 (7.3)	2 (5.1)	4 (4.9)	5 (16.7)	0.141
		High-normal	74 (49.0)	16 (41.0)	43 (52.4)	15 (50.0)	
		Hypertension	66 (43.7)	21 (53.8)	35 (42.7)	10 (33.3)	
	Baseline DBP category (old recommendations)	Normotension	67 (44.4)	14 (35.9)	35 (42.7)	18 (60.0)	0.258
		High-normal	18 (11.9)	4 (10.3)	12 (14.6)	2 (6.7)	
		Hypertension	66 (43.7)	21 (53.8)	35 (42.7)	10 (33.3)	
	SBP category at follow-up (new recommendations)	Normotension	18 (11.9)	7 (17.9)	7 (8.5)	4 (13.3)	0.606
		High-normal	108 (71.5)	26 (66.7)	62 (75.6)	20 (66.7)	
		Hypertension	25 (16.6)	6 (15.4)	13 (15.9)	6 (20.0)	
	SBP category at follow-up (old recommendations)	Normotension	64 (42.4)	17 (43.6)	35 (42.7)	12 (40.0)	0.987
		High-normal	62 (41.1)	16 (41.0)	34 (41.5)	12 (40.0)	
		Hypertension	25 (16.6)	6 (15.4)	13 (15.9)	6 (20.0)	
	DBP category at follow-up (new recommendations)	Normotension	10 (6.6)	1 (2.6)	6 (7.3)	3 (10.0)	0.241
		High-normal	129 (85.4)	32 (82.1)	71 (86.6)	26 (86.7)	
		Hypertension	12 (7.9)	6 (15.4)	5 (6.1)	1 (3.3)	
	DBP category at follow-up (old recommendations)	Normotension	99 (65.6)	25 (64.1)	52 (63.4)	22 (73.3)	0.253
		High-normal	40 (26.5)	8 (20.5)	25 (30.5)	7 (23.3)	
		Hypertension	12 (7.9)	6 (15.4)	5 (6.1)	1 (3.3)	
	SBP category decreased (new recommendations)	Yes	47 (31.1)	10 (25.6)	29 (35.4)	8 (26.7)	0.469
	SBP category decreased (old recommendations)	Yes	58 (38.4)	15 (38.5)	35 (42.7)	8 (26.7)	0.304
	DBP category decreased (new recommendations)	Yes	61 (40.4)	17 (43.6)	35 (42.7)	9 (30.0)	0.430
	DBP category decreased (old recommendations)	Yes	62 (41.1)	18 (46.2)	34 (41.5)	10 (33.3)	0.559
Comorbidities	ICD 10 category	C	5 (3.3)	3 (7.7)	1 (1.2)	1 (3.3)	0.177
		D	20 (13.2)	2 (5.1)	14 (17.1)	4 (13.3)	0.194
		E	84 (55.6)	24 (61.5)	42 (51.2)	18 (60.0)	0.489
		F	24 (15.9)	7 (17.9)	8 (9.8)	9 (30.0)	**0.032**
		G	17 (11.3)	7 (17.9)	8 (9.8)	2 (6.7)	0.277
		H	6 (4.0)	0 (0.0)	4 (4.9)	2 (6.7)	0.307
		I	112 (74.2)	25 (64.1)	64 (78.0)	23 (76.7)	0.246
		J	25 (16.6)	10 (25.6)	10 (12.2)	5 (16.7)	0.177
		K	71 (47.0)	14 (35.9)	46 (56.1)	11 (36.7)	0.051
		L	8 (5.3)	2 (5.1)	5 (6.1)	1 (3.3)	0.845
		M	86 (57.0)	24 (61.5)	46 (56.1)	16 (53.3)	0.771
		N	11 (7.3)	2 (5.1)	5 (6.1)	4 (13.3)	0.356
		R	38 (25.2)	8 (20.5)	20 (24.4)	10 (33.3)	0.464
		T	16 (10.6)	5 (12.8)	7 (8.5)	4 (13.3)	0.668
		Z	80 (53.0)	23 (59.0)	45 (54.9)	12 (40.0)	0.258
Medical actions/interventions	Dosage modified for existing drug	Yes	122 (80.8)	32 (82.1)	65 (79.3)	25 (83.3)	0.866
	Expanded diagnostics	Yes	47 (31.1)	12 (30.8)	26 (31.7)	9 (30.0)	0.984
	Introduced new drug	Yes	123 (81.5)	32 (82.1)	67 (81.7)	24 (80.0)	0.973
	Issue dietary recommendations	Yes	99 (65.6)	25 (64.1)	56 (68.3)	18 (60.0)	0.698
	New diagnosis	Yes	54 (35.8)	14 (35.9)	31 (37.8)	9 (30.0)	0.747
	Referral to hospital	Yes	3 (2.0)	1 (2.6)	1 (1.2)	1 (3.3)	0.743
	Referral to hospital of specialist clinic	Yes	62 (41.1)	14 (35.9)	35 (42.7)	13 (43.3)	0.747
	Referral to specialist clinic	Yes	60 (39.7)	13 (33.3)	34 (41.5)	13 (43.3)	0.628
	Treatment of recurrent condition	Yes	29 (19.2)	4 (10.3)	22 (26.8)	3 (10.0)	**0.035**

Values are given as counts (frequency [%]).

No strong linear trends were detected across the three polypharmacy categories for age, anthropometric measures, or laboratory parameters (all p > 0.05). Median age increased slightly from 47 years (IQR 44–50) in the ≤5-drug group to 49 years (IQR 46–51) in the ≥7-drug group, yet this shift did not reach statistical significance (HL = −1.0; 95% CI, −3.0 to 1.0; p = 0.22). Similarly, systolic and diastolic blood pressure, lipid markers (non-HDL-C, TG), fasting glucose, and SCORE2 estimates showed no monotonic association with higher medication counts. The Hodges–Lehmann estimates for differences between the lowest and highest polypharmacy categories were all small in magnitude, and their 95% confidence intervals crossed zero, indicating absence of systematic location shifts between distributions. These findings confirm that the subsequent multivariable models explore subtle, multidimensional co-variation rather than simple univariate gradients.

Among categorical variables, only a few domain-specific patterns reached formal statistical significance after trend or χ^2^ testing. Patients treated in earlier years of data collection showed a marginally higher prevalence in the lowest medication category (p = 0.031). Irregular heart rhythm at baseline was more frequent in patients with ≥7 drugs (30.0%) than in those with ≤5 drugs (12.8%, p = 0.007), and transitions from irregular to regular rhythm during follow-up also concentrated within this group (p = 0.001). Similarly, treatment for recurrent conditions was more often recorded among patients with six or more concurrent drugs (26.8% vs. 10.3%; p = 0.035). All other demographic, behavioral, and comorbidity indicators—including sex, domicile, education, smoking, alcohol use, coffee intake, and ICD-10-based disease categories—showed no statistically significant variation across the polypharmacy strata (all p > 0.05).

### 3.2 Insights into multivariate characteristics in context of polypharmacy

Because both feature-selection strategies (forward and backward) converged on the same subset of predictors in Models A and B, the distinction between them became redundant. For clarity, the results are henceforth reported as *multimorbidity-adjusted models* differing only by their selection approach (forward vs. backward).

Both adjusted models demonstrated satisfactory and stable goodness-of-fit (Supplementary Material, Supplementary Table S5). In the forward-selected model, the log-likelihood improved from −151.3 (naïve intercept-only model) to −139.9, yielding a likelihood-ratio statistic of 22.7 (df = 4; p < 0.001). The backward-selected model achieved an even higher log-likelihood of −136.1 (LR = 30.6; df = 8; p < 0.001). Corresponding McFadden pseudo-R^2^ values ranged from 0.075 to 0.10, consistent with moderate explanatory strength given the limited sample size (N = 151). Information criteria were closely aligned across models (AIC ≈292; BIC ≈315–322), suggesting stable behavior under alternative specifications. Collectively, these indices support the internal consistency of both multimorbidity-adjusted solutions, without clear indications of model overfitting, though external validation would be required to confirm their generalizability.

In both multimorbidity-adjusted proportional-odds models (Table 3), consistent associations emerged across the forward- and backward-selected solutions. The multimorbidity count, retained solely as an adjustment covariate, did not show a meaningful direct effect (p = 0.46–0.99). In the forward-selected model, higher odds of being in a higher polypharmacy category were observed among patients who showed a transition from irregular to regular heart rhythm at follow-up (OR = 6.0; 95% CI, 1.83–19.9; p = 0.003) and among those with circulatory diseases (ICD-10 I) (OR = 2.28; 95% CI, 1.05–4.95; p = 0.037). In contrast, the presence of nervous system diseases (ICD-10 G) was inversely associated with polypharmacy (OR = 0.33; 95% CI, 0.11–0.95; p = 0.039), and higher baseline diastolic blood pressure (DBP) showed a modest negative association (OR per 1 mmHg = 0.95; 95% CI, 0.93–0.98; p = 0.002).

**TABLE 3:** Multimorbidity-adjusted multivariate models exploring the association of selected patient features and the odds of higher medication burden, categorized based on concurrent drug usage count: 5, 6, and ≥7.

Multimorbidity-adjusted model derived from forward stepwise feature selection; modeling the odds of being in a higher polypharmacy category								
Variable (term)	exp (β_i_) interpretation	β_i_	β_i_ SE	t	p	exp (β_i_)	exp (β_i_) LCI	exp (β_i_) UCI
multicomorbidity_count	(Covariate— not interpretable in context of this model)	−0.0024	0.2424	−0.0101	0.992	0.998	0.620	1.604
Heartrhythmchange_ irregulartoregular1	OR between patients who showed change from regular to irregular HR at follow-up, and those in whom the change was not observed	1.7950	0.6090	2.9473	**0.003**	6.020	1.825	19.861
dbp_0	OR per every 1 mmHg increase of baseline DBP	−0.0467	0.0147	−3.1674	**0.002**	0.954	0.927	0.982
icd10_I1	OR between patients with circulatory diseases, and those without them	0.8244	0.3953	2.0855	**0.037**	2.281	1.051	4.949
icd10_G1	OR between patients with nervous system diseases, and those without them	−1.1143	0.5398	−2.0645	**0.039**	0.328	0.114	0.945
multicomorbidity_count	(Covariate — not interpretable in context of this model)	−0.1774	0.2415	−0.7346	0.463	0.837	0.522	1.344
education22	OR between patients declaring higher education, and those of lower education	0.7214	0.3789	1.9041	0.057	2.057	0.979	4.323
dbp_0	OR per every 1 mmHg increase of baseline DBP	−0.0762	0.0255	−2.9879	**0.003**	0.927	0.881	0.974
dbp_change	OR per every 1 mmHg higher DBP at follow-up compared to the baseline values	−0.0573	0.0342	−1.6732	0.094	0.944	0.883	1.010
fg_change	OR per every 1 mg/dL increase in fasting glucose during follow-up compared to the baseline values	−0.0166	0.0076	−2.1944	**0.028**	0.984	0.969	0.998
Heartrhythmchange_ irregulartoregular1	OR between patients who showed change from regular to irregular HR at follow-up, and those in whom the change was not observed	2.1281	0.6221	3.4205	**0.001**	8.399	2.481	28.430
icd10_H1	OR between patients with sensory (eye/ear) diseases, and those without them	1.6257	0.7983	2.0365	**0.042**	5.082	1.063	24.297
icd10_I1	OR between patients with circulatory diseases, and those without them	0.9453	0.4033	2.3441	**0.019**	2.573	1.168	5.672

All odds ratios are adjusted for the multimorbidity count, which was retained as a covariate for comparability but is not interpreted directly. Original variable (term) names are retained in this table so as to align with the documentation featured in Supplementary Material; their meaning is given in the ‘Interpretation’ column. LCI, and UCI, lower and upper limit of the 95% confidence interval; OR, odds ratio; SE, estimate standard error. Intercepts from the model are not shown since they are not directly interpretable.

The backward-selected model reproduced these general patterns while expanding the predictor set. Associations persisted for circulatory disease (ICD-10 I; OR = 2.57; 95% CI, 1.17–5.67; p = 0.019) and rhythm normalization at follow-up (OR = 8.40; 95% CI, 2.48–28.4; p = 0.001). Additionally, higher education was weakly linked to increased odds of polypharmacy (OR = 2.06; 95% CI, 0.98–4.32; p = 0.057). Small but directionally coherent negative associations were noted for baseline DBP (OR per 1 mmHg = 0.93; 95% CI, 0.88–0.97; p = 0.003), follow-up DBP relative to baseline (OR = 0.94; 95% CI, 0.88–1.01; p = 0.094), and fasting glucose change (OR = 0.98; 95% CI, 0.97–1.00; p = 0.028). The sensory disease category (ICD-10 H), encompassing ophthalmologic and otologic conditions, also showed a positive relation to higher polypharmacy (OR = 5.08; 95% CI, 1.06–24.3; p = 0.042).

### 3.3 Bootstrap-based stability of the multimorbidity-adjusted models

The bootstrap validation confirmed the internal consistency of both multimorbidity-adjusted models. Among the forward-selected models, all effects that were statistically significant in the original proportional-odds fit retained their sign and approximate magnitude under resampling. Robust associations—defined as those whose 95% bootstrap percentile interval did not cross zero—were observed for baseline diastolic blood pressure, change from irregular to regular heart rhythm at follow-up, and two ICD-10 domains: circulatory diseases (I) and nervous-system diseases (G). The intercept separating the highest polypharmacy category also remained stable, suggesting that the ordinal thresholds were not materially affected by resampling noise.

In the corresponding backward-selection model adjusted for multimorbidity, a similar pattern emerged. The same core predictors—baseline DBP, change from irregular to regular heart rhythm at follow-up, and ICD-10 I and H categories—displayed narrow bootstrap intervals entirely above or below zero, indicating directionally consistent effects. The education variable approached the robustness threshold, with its confidence limits narrowly spanning zero, whereas between-timepoint dynamics in DBP and FG showed wide, cross-zero intervals, suggesting less reliable contributions. Multimorbidity, included for adjustment only, consistently exhibited wide, non-significant percentile ranges, confirming its role as a control covariate rather than an explanatory factor.

**FIGURE 3:**
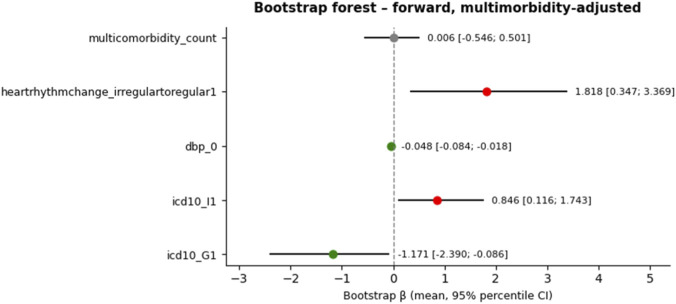
Bootstrap-derived estimates of regression coefficients (β) with 95% percentile confidence intervals from multimorbidity-adjusted proportional-odds model obtained via forward stepwise feature selection. Each point represents the mean bootstrap estimate across 200 nonparametric resamples, while horizontal bars indicate the 2.5th–97.5th percentile range. Red markers denote positive and statistically robust associations (confidence interval not crossing zero), green markers denote negative and robust associations, and gray markers correspond to uncertain or non-robust effects. For interpretability, intercept terms separating ordinal outcome thresholds (5|6 and 6|7plus) are omitted from the plots.

**FIGURE 4:**
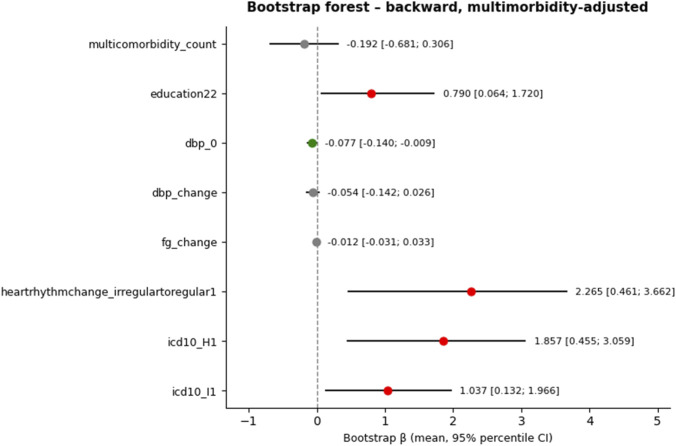
Bootstrap-derived estimates of regression coefficients (β) with 95% percentile confidence intervals from multimorbidity-adjusted proportional-odds model obtained via backward stepwise feature selection. Each point represents the mean bootstrap estimate across 200 nonparametric resamples, while horizontal bars indicate the 2.5th–97.5th percentile range. Red markers denote positive and statistically robust associations (confidence interval not crossing zero), green markers denote negative and robust associations, and gray markers correspond to uncertain or non-robust effects. For interpretability, intercept terms separating ordinal outcome thresholds (5|6 and 6|7plus) are omitted from the plots.

Overall, the bootstrap analysis supports the stability of the principal findings—particularly those related to blood-pressure measures, heart-rhythm normalization, and cardiovascular or sensory disease domains—while underscoring that weaker effects (education, glucose change) should be regarded as exploratory. The combination of tabular summaries (Supplementary Table S6) and coefficient-scale visualization (Figures 3, 4 for forward- and backward-derived models, respectively) highlights the moderate but reproducible structure of associations captured by the multimorbidity-adjusted ordinal models.

## 4 Discussion

In this sub-study of middle-aged adults enrolled in the Prophylaxis 40 PLUS programme, we observed that polypharmacy was highly prevalent and strongly patterned by cardiovascular morbidity, rhythm-control interventions, sensory comorbidities, and educational attainment. These patterns support the view that, in a preventive-care context, polypharmacy functions as a composite marker of therapeutic and organisational complexity rather than a simple proxy for disease count (Masnoon et al., 2017; Guillot et al., 2020; Skou et al., 2022). The most consistent predictor of being in a higher polypharmacy stratum was the presence of circulatory system disease (ICD-10 I). This is not surprising: individuals with hypertension, ischaemic heart disease, arrhythmias, or other cardiovascular conditions typically receive multi-component pharmacotherapy, including blood-pressure–lowering agents, lipid-lowering therapy, antiplatelet or anticoagulant drugs, and sometimes anti-anginal or anti-arrhythmic medications (Banach et al., 2025; Visseren et al., 2021; Badimon et al., 2023). In our data, the association between circulatory diagnoses and medication count likely reflects guideline-directed intensification of preventive therapy, consistent with ESC recommendations for individuals at elevated cardiovascular risk (Visseren et al., 2021). From this perspective, at least part of the observed polypharmacy can reasonably be considered appropriate, as it aligns with evidence-based efforts to reduce long-term cardiovascular events (Banach et al., 2025; Badimon et al., 2023; Kotseva et al., 2019).

We also found that normalisation of previously irregular heart rhythm during follow-up was associated with higher polypharmacy levels. This pattern suggests that patients undergoing rhythm-control strategies—whether pharmacological, device-based, or procedural—are exposed to intensified cardiovascular pharmacotherapy, including rate- or rhythm-controlling drugs and anticoagulation where indicated (Visseren et al., 2021; Varma et al., 2021). Digital tools and remote monitoring may further expand treatment options and detection of arrhythmias, potentially increasing both the benefits and complexity of therapy (Widmer et al., 2015; Varma et al., 2021). In the prevention-programme setting, this underscores the need for coordinated medication review whenever rhythm status changes, both to ensure ongoing indication and to mitigate risks such as bleeding or pro-arrhythmia. It is important to note, however, that “normalisation of previously irregular heart rhythm” may represent a clinical outcome of treatment rather than an independent predictor. The possibility of reverse causality whereby rhythm normalisation is itself a consequence of intensified pharmacotherapy rather than a driver of polypharmacy cannot be excluded in this cross-sectional analytical context, and this interpretation should be treated with appropriate caution.

Conversely, we observed an inverse association between polypharmacy and baseline diastolic blood pressure, with lower diastolic values seen in individuals with higher medication counts. This likely reflects the downstream effect of more aggressive antihypertensive treatment patients on multiple cardiovascular medications achieve lower blood-pressure levels, consistent with the treatment targets recommended by ESC and other medical societies (Banach et al., 2025; Visseren et al., 2021). While such control is generally beneficial for long-term risk reduction, it also raises concerns about overtreatment and symptomatic hypotension, particularly in multimorbid patients or those with impaired autoregulation (Roth et al., 2020; Skou et al., 2022). These observations reinforce the need for careful balance between treatment intensity and tolerability, especially when multiple agents are used.

An interesting and somewhat contrasting pattern was the negative association between polypharmacy and nervous-system disease (ICD-10 G). One plausible interpretation is prescribing restraint in patients with neurological conditions such as epilepsy, stroke-related cognitive impairment, or neurodegenerative disorders. Clinicians may be more cautious about adding further medications in these groups because of concerns about central nervous system adverse effects, interactions with antiepileptic or psychotropic drugs, and potential impact on cognition and function (Maher et al., 2014; Khezrian et al., 2020; Nishtala and Salahudeen, 2015; Zhao et al., 2023). Previous work has shown that neurological and psychiatric comorbidities are often associated with both higher risk of medication-related problems and greater uncertainty about optimal prescribing (Khezrian et al., 2020; Ye et al., 2022; Kurczewska-Michalak et al., 2021). Our findings suggest that, in a prevention-programme setting, this may sometimes manifest as lower overall medication counts, which could reflect either appropriate simplification or, in some cases, undertreatment of cardiovascular risk.

We also found that sensory system diseases (ICD-10 H) were associated with higher levels of polypharmacy. Although ICD-10 H covers a diverse range of ophthalmologic and otologic conditions, its association with medication burden likely reflects the cumulative effect of multi-specialty care. As patients engage with additional specialists—for example for glaucoma, retinal disease, or chronic otitis—they accrue further prescriptions (topical and systemic), adding complexity to already dense regimens initiated in primary care (Guillot et al., 2020; Maher et al., 2014; Davies et al., 2020; Kurczewska-Michalak et al., 2021). This pattern aligns with literature showing that the number of prescribers and care transitions is a key driver of polypharmacy and potentially inappropriate medications, particularly in multimorbid older adults (Kurczewska-Michalak et al., 2021; Gnjidic et al., 2012; Rankin et al., 2018; Roncal-Belzunce et al., 2024).

Socio-demographic factors also played a role. In our cohort, higher educational attainment tended to be associated with greater odds of polypharmacy, though the effect was modest. This finding is consistent with research suggesting that individuals with higher education may have greater health literacy, are more likely to engage with preventive services, and may be more willing to accept intensification of therapy when recommended (Cho et al., 2022; Kardas et al., 2021; Kim and Parish, 2017). However, it also raises potential equity concerns: if less-educated individuals receive fewer guideline-directed therapies despite similar risk profiles, this could contribute to disparities in long-term outcomes (Roth et al., 2020; Nicholson et al., 2024; Kardas et al., 2021; Skou et al., 2022). For national programmes like Prophylaxis 40 PLUS, it will be important to monitor whether preventive pharmacotherapy is offered and accepted equitably across education levels.

From a methodological perspective, we used ordinal proportional-odds regression to examine how various characteristics align with increasing polypharmacy, treating medication count as a structured, ordered outcome rather than a binary variable. This approach is well suited to outcomes with natural categories (e.g., 5, 6, ≥7 drugs) and allows efficient use of information distributed across these levels [38]. It complements traditional regression or dichotomised analyses that risk obscuring meaningful differences between moderate and very high medication burdens. Our use of a pre-specified modelling strategy, adjustment for multimorbidity, and bootstrap-based assessment of stability is in line with recommendations for analysing complex, multidimensional outcomes (Delara et al., 2022; Skou et al., 2022; Rankin et al., 2018; Wang et al., 2024).

Our findings fit within the broader literature on polypharmacy, multimorbidity, and prevention. Systematic reviews have documented strong associations between polypharmacy and adverse outcomes, but also highlight that the context of prescribing—such as guideline-driven preventive therapy versus uncontrolled therapeutic drift—is critical for interpretation (Davies et al., 2020; Khezrian et al., 2020; Pazan and Wehling, 2021; Delara et al., 2022; Pazan and Wehling, 2017; Maher et al., 2014). Evidence from polypharmacy-management interventions indicates that structured medication reviews, explicit criteria (such as FORTA), and multidisciplinary collaboration can reduce inappropriate medications and improve prescribing quality, even if effects on mortality or hospitalisation are modest (Kurczewska-Michalak et al., 2021; Pazan and Wehling, 2017; Rankin et al., 2018; Jungo et al., 2023; Roncal-Belzunce et al., 2024). Our results suggest that such strategies could be fruitfully embedded in cardiovascular prevention programmes, where polypharmacy is highly prevalent, but much of it may represent necessary, risk-reducing therapy. In particular, individuals with circulatory disease and those undergoing arrhythmia-related interventions might benefit from targeted, programme-integrated medication review, rather than relying solely on opportunistic adjustments during routine visits.

The role of digital health deserves special mention. Remote monitoring, mobile health applications, and teleconsultations have been shown to support risk-factor control and adherence in CVD prevention (Widmer et al., 2015). In arrhythmia management, digital tools can enhance detection, follow-up, and therapy optimisation, but may also lead to more frequent medication changes and potential escalation of treatment complexity (Varma et al., 2021). Integrating digital solutions with structured medication-review workflows could help ensure that pharmacotherapy intensified on the basis of digital signals remains coherent, safe, and patient-centred.

### 4.1 Study limitation

This study has several limitations. First, the sample size was modest, limiting the precision of estimates and precluding detailed exploration of interactions between specific comorbidities or drug classes. Second, the observational design means that causal relationships cannot be inferred. Third, we did not apply explicit criteria (e.g., FORTA or other tools) to distinguish appropriate from potentially inappropriate medications, so our measure of polypharmacy captures quantity but not quality of prescribing (Kurczewska-Michalak et al., 2021; Pazan and Wehling, 2017). Fourth, our cohort was drawn from a single national programme and may not be fully representative of all Prophylaxis 40 PLUS participants or of middle-aged adults in other settings. Fifth, information on treatment adherence, indication strength, and clinical decision-making processes was not available. Nonetheless, the structured nature of the programme and the consistent associations seen across modelling approaches support the internal validity and relevance of the findings. Finally, the analysed age range represents a restricted segment of middle-aged adults, which may limit extrapolation to older populations or to the full spectrum of programme participants. Despite these limitations, the structured data collection and consistent findings across modelling approaches support the internal validity of the results. Sixth, and importantly, the final analytical sample of 151 participants represents a highly selected subgroup drawn from a much larger pool of 2,572 distinct programme participants. The requirement for simultaneous fulfilment of both polypharmacy and multimorbidity criteria substantially narrows the study population and limits the generalisability of the findings to the broader Prophylaxis 40 PLUS cohort. This selection should be taken into account when interpreting the results. Seventh, medication data were analysed as counts rather than by therapeutic class; consequently, the specific drug classes driving the observed associations particularly in relation to circulatory disease—cannot be directly verified from the available data. Future studies should include detailed medication-class data to better characterise the nature and appropriateness of polypharmacy in this population. Eighth, the variable “normalisation of previously irregular heart rhythm” was included as a predictor in the regression models; however, it may also represent a clinical outcome of treatment rather than an independent driver of medication burden. Reverse causality cannot be excluded, and this variable should be interpreted with caution.

### 4.2 Implications for practice and policy

Within a structured cardiovascular prevention programme, polypharmacy appears to function primarily as a marker of therapeutic intensity and system-level complexity rather than as a simple reflection of disease count. The strong association between higher medication burden and circulatory system disease suggests that much of the observed polypharmacy reflects guideline-directed preventive care. In this context, polypharmacy should not be interpreted automatically as inappropriate, but as a signal warranting clinical attention. Our findings indicate that certain clinical transitions may represent key moments for medication review. In particular, normalization of previously irregular heart rhythm was strongly linked to higher polypharmacy, suggesting that rhythm-control interventions are points at which pharmacotherapy is often intensified and restructured. Embedding structured medication review at such transition points could help maintain therapeutic coherence and safety. The inverse association between baseline diastolic blood pressure and medication burden further highlights the need to balance aggressive risk-factor control with tolerability, especially in patients receiving multiple cardiovascular agents. Differences across comorbidity domains point to distinct prescribing dynamics. Lower medication counts in patients with nervous-system diseases may reflect cautious prescribing, whereas higher polypharmacy in those with sensory-system conditions likely reflects multispecialty care. Finally, the tendency toward higher medication burden among better-educated participants raises questions about equity in access to preventive pharmacotherapy. Together, these observations support integrating targeted, transition-focused medication review into national cardiovascular prevention pathways.

## 5 Conclusion

In middle-aged adults participating in a national cardiovascular prevention programme, polypharmacy appears to act as a dynamic indicator of therapeutic intensity and healthcare system complexity rather than as a simple consequence of multimorbidity alone. Higher medication burden was consistently associated with circulatory system disease, rhythm-control–related clinical transitions, and selected comorbidity domains, while overall multimorbidity showed limited explanatory value. These findings suggest that, within preventive-care pathways, polypharmacy reflects patterns of guideline-directed treatment intensification and multispecialty involvement. Recognising polypharmacy as an ordered, system-level marker may help to identify clinical contexts in which structured medication review could be particularly relevant, especially during key therapeutic transitions. Future research should explore how such approaches can be integrated into prevention programmes while maintaining a balance between effective risk reduction and medication safety.

## Data Availability

The raw data supporting the conclusions of this article will be made available by the authors, without undue reservation.
